# Replication methods and tools in high-throughput cultivation processes - recognizing potential variations of growth and product formation by on-line monitoring

**DOI:** 10.1186/1472-6750-10-22

**Published:** 2010-03-16

**Authors:** Robert Huber, Thomas G Palmen, Nadine Ryk, Anne-Kathrin Hillmer, Karina Luft, Frank Kensy, Jochen Büchs

**Affiliations:** 1 AVT - Aachener Verfahrenstechnik, Biochemical Engineering, RWTH Aachen University, Worringerweg 1, D-52074 Aachen, Germany; 2 m2p-labs GmbH, Forckenbeckstr. 6, 52074 Aachen, Germany

## Abstract

**Background:**

High-throughput cultivations in microtiter plates are the method of choice to express proteins from recombinant clone libraries. Such processes typically include several steps, whereby some of them are linked by replication steps: transformation, plating, colony picking, preculture, main culture and induction. In this study, the effects of conventional replication methods and replication tools (8-channel pipette, 96-pin replicators: steel replicator with fixed or spring-loaded pins, plastic replicator with fixed pins) on growth kinetics of *Escherichia coli *SCS1 pQE-30 pSE111 were observed. Growth was monitored with the BioLector, an on-line monitoring technique for microtiter plates. Furthermore, the influence of these effects on product formation of *Escherichia coli *pRhotHi-2-EcFbFP was investigated. Finally, a high-throughput cultivation process was simulated with *Corynebacterium glutamicum *pEKEx2-phoD-GFP, beginning at the colony picking step.

**Results:**

Applying different replication tools and methods for one single strain resulted in high time differences of growth of the slowest and fastest growing culture. The shortest time difference (0.3 h) was evaluated for the 96 cultures that were transferred with an 8-channel pipette from a thawed and mixed cryoculture and the longest time difference (6.9 h) for cultures that were transferred with a steel replicator with fixed pins from a frozen cryoculture. The on-line monitoring of a simulated high-throughput cultivation process revealed strong variances in growth kinetics and a twofold difference in product formation. Another experiment showed that varying growth kinetics, caused by varying initial biomass concentrations (OD_600 _of 0.0125 to 0.2) led to strongly varying product formation upon induction at a defined point of time.

**Conclusions:**

To improve the reproducibility of high-throughput cultivation processes and the comparability between different applied cultures, it is strongly recommended to use automated or manual liquid handling stations or, alternatively, multi-channel pipettes. Because of their higher transfer volume and hence precision in comparison to pin replicators, they reduce the variance of initial biomass concentrations. With respect to the results obtained, other methods to increase the comparability between parallel cultivations by compensating differences in biomass concentrations are required, such as using autoinduction media, fed-batch operation of precultures or on-line monitoring in microtiter plates combined with automated liquid handling.

## Background

In recent years, many advances have been made in biomolecular disciplines for the genetic manipulation of prokaryotes and eukaryotes, such as high-throughput cloning techniques [1-3]. This leads to an increasing availability and utilization of genetically different clones (clone libraries). Nowadays, one of the most important purposes of clone libraries is to produce recombinant proteins for different applications (e.g. structural proteomics, protein arrays). This is mostly conducted with *E. coli *expression systems. Clone libraries are normally cultivated in parallel high-throughput cultivation processes. For that purpose, microtiter plates (MTPs) are predominantly used because of their high parallelism, low costs and easy handling [4-9]. Such MTP-based processes mostly consist of multiple cultivation steps [4,10,11]. The first step is to establish the clone library, mainly via transformation of competent *E. coli *cells with plasmids harboring different target genes. Then, the transformation mix is normally plated on selective agar plates. In a next step, single clones from that agar plates are picked and transferred to a preculture-MTP. Sometimes the transformation mix is also directly applied to the precultivation step. Even though the cryopreservation of *E. coli *might lead to a loss of viability [12,13] and product yield [13], often cryocultures are established from the preculture-MTP, as it can be impractical to prepare a fresh transformation mix for each expression study. In summary, there are three possible sources that are used as starting material for high-throughput production processes for recombinant proteins: cryocultures, freshly transformed cells or single colonies from agar plates. The next step is to use this starting material in a preculture-MTP, followed by a main cultivation.

In the main culture, target protein expression is mostly induced by adding an inducing substance to the cultivation medium such as isopropyl β-D-1-thiogalactopyranoside (IPTG). This induction takes place at a defined optical density (OD) [10] or, more often, at a defined point of time [10,14-16]. Besides this conventional method of inducing cultures the autoinduction principle is also applied, which uses a specially designed medium [17]. Here, the induction is triggered automatically for T7 *E. coli *expression systems by lactose when the other carbon source (glucose) in the medium is exhausted, thus circumventing the effect of induction at different growth phases. To sum up, a typical high-throughput production process for a clone library mostly consists of seven unit operations: transformation, plating, colony picking, preculture, main culture, induction and harvest. This high number of steps might increase the risk of errors. Although there are new methods emerging that try to simplify this complex workflow [18], the described process can be considered as state-of-the-art. To combine the cultivation steps of such a process, a transfer of clones from a source MTP to a target MTP is necessary, which is mainly conducted with replicator tools for liquid transfer. Easy to handle tools are for example 96-pin replicators (manufactured as a disposable or as reusable steel variant) [15,19]. Dependent on the dimensions and characteristics (e. g. coating) of the pins, their transfer volume lies in a range of only few nanoliters to several microliters. Moreover, liquid handling stations with multi-channel pipette heads (automated or manually operated) are applied for replicating clones [20]. Interestingly, the influence of the replication step on growth and protein expression in MTP-based high-throughput processes has not been addressed in dedicated investigations so far. Although the limits of the different replication techniques might be known, the awareness for problems that can arise due to replication steps is quite low. A possible reason for that is probably the lack of appropriate monitoring tools for cultivations in MTPs [21,22]. With the BioLector-technique it is possible to permanently monitor growth and fluorescence of reporter proteins in a MTP. In contrast to a MTP-reader, the BioLector measurement is conducted without interrupting the shaking motion, thus not interfering with the oxygen supply of the cultures [21,23].

Therefore, the aim of this study was to use the BioLector device to quantify for the first time the effects of different replication tools and replication methods on the cultivation of an *E. coli *strain (SCS1 pQE-30 pSE111) in MTPs. The influence of these effects on product formation was studied with the *E. coli *strain pRhotHi-2-EcFbFP, which expresses a fluorescence protein upon induction. Furthermore, the typical process flow from colony picking, preculture and cryopreservation to protein induction at a defined point of time was studied in respect to growth kinetics and product formation. For that purpose, the strain *Corynebacterium glutamicum *pEKEx2-phoD-GFP was applied.

## Methods

### Microorganism

The strain *E. coli *SCS1 pQE-30 pSE111 (here referred to as *E. coli *PR02) [11] was used for the replicator tool and replication method experiments. It was kindly provided by Protagen AG, Dortmund, Germany. To prepare stock cultures, a preculture in TB medium was made. After reaching the stationary phase (OD_600 _= 22), an equal amount of glycerol solution (500 g/L) was added as a cryoprotectant, resulting in a final glycerol concentration of 250 g/L and a final OD_600 _of 11. The cultures were stored at -20°C in 100 μL aliquots in 96-well MTPs, which were closed with an adhesive seal (Cat. AB-0580, Thermo Scientific, Karlsruhe, Germany).

The clone *E. coli *BL21(DE3) pRhotHi-2-EcFbFP [24] was used to investigate the influence of different initial optical densities on product formation upon induction at a defined point of time. As a T7 expression system, this strain produces the fluorescence protein EcFbFP [25] when induced with IPTG. This strain was kindly provided by T. Drepper, Institute of Molecular Enzyme Technology, Heinrich-Heine-University Düsseldorf, Germany. To prepare stock cultures, a preculture in TB medium was made. After reaching the stationary phase, fresh medium and glycerol solution (500 g/L) were added as a cryoprotectant, resulting in a final glycerol concentration of 150 g/L and a final OD_600 _of 4. The cultures were stored at -20°C in 1 mL aliquots in cryo-vials.

The strain *Corynebacterium glutamicum *pEKEx2-phoD-GFP [26] was used for the experiments to simulate a high-throughput cultivation process and was kindly provided by R. Freudl, Institute of Biotechnology 1, Forschungszentrum Jülich, Germany. The transformed colonies were provided on agar plates (modified Eikmanns mineral medium with additional 16 g/L agar). Upon induction with IPTG, this strain produces and secretes the green fluorescence protein (GFP).

### Media

Terrific Broth (TB) medium was used for all *E. coli *PR02 cultivations. It consists of 5 g/L glycerol, 12 g/L tryptone, 24 g/L yeast extract, 12.54 g/L K_2_HPO_4 _and 2.31 g/L KH_2_PO_4_. The pH was adjusted to 7.2 with NaOH. Additionally, 0.1 g/L ampicillin was added.

*E. coli *BL21(DE3) pRhotHi-2-EcFbFP was cultivated in MDG medium which was prepared as described by Studier [17]. It consists of 3.55 g/L Na_2_HPO_4_, 3.4 g/L KH_2_PO_4_, 2.675 g/L NH_4_Cl, 0.71 g/L Na_2_SO_4_, 0.493 g/L MgSO_4_*7H_2_O, 0.365 mg/L FeCl_3_*6H_2_O, 0.444 mg/L CaCl_2_, 0.396 mg/L MnCl_2_*4H_2_O, 0.575 mg/L ZnSO_4_*7H_2_O, 0.095 mg/L CoCl_2_*6H_2_O, 0.068 mg/L CuCl_2_*2H_2_O, 0.095 mg/L NiCl_2_*6H_2_O, 0.097 mg/L Na_2_MoO_4_*4H_2_O, 0.105 mg/L Na_2_SeO_3_*5H_2_O, 0.025 mg/L H_3_BO_3_, 2.5 g/L aspartate and 5 g/L glucose. The pH was adjusted to 6.8 with NaOH. Additionally, 0.05 g/L kanamycin was added to the medium.

A modified Eikmanns mineral medium [27] was used for the *C. glutamicum *pEKEx2-phoD-GFP cultivations. It consists of 10 g/L glucose, 10 g/L (NH_4_)_2_SO_4_, 1 g/L KH_2_PO_4_, 2 g/L K_2_HPO_4_, 0.25 g/L MgSO_4_*7H_2_O, 21 g/L MOPS (C_7_H_15_NO_4_S), 0.2 mg/L biotin, 10 mg/L CaCl_2_, 30 mg/L protocatechuic acid, 10 mg/L FeSO_4_*7H_2_O, 0.2 mg/L CuSO_4_, 10 mg/L MnSO_4_*H_2_O, 0.02 mg/L NiCl_2_*6H_2_O, 1 mg/L ZnSO_4_*7H_2_O. The pH was adjusted to pH 7.2 with NaOH. Additionally, 0.025 g/L kanamycin was added to the medium.

All reagents were purchased from Merck (Darmstadt, Germany), Carl Roth (Karlsruhe, Germany) or Sigma Aldrich (Taufkirchen, Germany).

### BioLector

The *E. coli *PR02 and *C. glutamicum *cultivations were conducted in a BioLector prototype, corresponding to the description by Samorski et al. [21]. The experiments with *E. coli *pRhotHi-2-EcFbFP were conducted with a commercial BioLector device (m2p-labs GmbH, Aachen, Germany) [23]. To detect microbial growth, light with a wavelength of 620 nm is sent into each culture well of a MTP from the bottom and the backscattered light intensity is detected again from the bottom. The EcFbFP fluorescence of the *E. coli *pRhotHi-2-EcFbFP cultivations was measured at an excitation of 460 nm and an emission of 492 nm, the GFP fluorescence of the *C. glutamicum *cultivations was measured at an excitation of 485 nm and an emission of 520 nm. The cultivations were carried out in black 96-well MTPs with a clear bottom (μclear, Greiner Bio-one, Frickenhausen, Germany) which were sealed with adhesive gas permeable seals (Cat. AB-0718, Thermo Scientific). All cultures were grown at 37°C, a shaking frequency of 950 rpm (*E. coli *PR02 and *E. coli *BL21(DE3) pRhotHi-2-EcFbFP) or 995 rpm (*C. glutamicum *pEKEx2-phoD-GFP), a shaking diameter of 3 mm and a filling volume of 200 μL per well. The experimental data were standardized to eliminate factors that influence the scattered light intensity (I) such as media background, type of MTP or geometrical positioning [21]. Hence, the initial value of scattered light intensity (I_0_) was subtracted from every value (I-I_0_). The same has been done for the fluorescence signal.

### Replication tools and experiments

Four replication tools were applied: an 8-channel pipette (Research 5 μL-100 μL, Eppendorf AG, Hamburg, Germany) to mimic a liquid handling system (e. g. 96-channel pipetting head) and three different pin tools. First, a disposable plastic replicator with fixed pins (QRep 96 pin, Genetix GmbH, München, Germany) made from polypropylene, second, a reusable steel replicator with fixed pins (96-pin replicator, Boekel scientific, Feasterville, USA) and third, a reusable steel replicator with 96 spring-loaded pins that may move vertically, dependent on the surface of the frozen liquid of a cryoculture-MTP (Duetz replicator system, EnzyScreen BV, Leiden, Netherlands) [28].

The disposable plastic replicator and the reusable steel replicator with fixed pins were used to inoculate target MTPs filled with 200 μL of TB medium with cells from thawed and mixed *E. coli *PR02 cryoculture-MTPs. As the lowest volume that can be transferred with the applied 8-channel pipette is 5 μL and the transfer volume of the pin replicators was lower than 1 μL, a dilution (1:10) with TB medium was made for the 8-channel pipette in order to achieve comparable initial biomass concentrations in the main culture.

In further experiments, the steel replicator with fixed pins was applied to inoculate target MTPs with cells from a frozen cryoculture-MTP or from the same cryoculture-MTP after thawing without mixing. Replication with the steel replicator with spring-loaded pins was also conducted with a frozen cryoculture-MTP. Thus, the pins of the replicator were put into the wells of a cryoculture-MTP with light pressure for three seconds so that the pins came into contact with the frozen liquid. Thereafter, the pins were put into the wells of the target MTP filled with 200 μL TB medium. After these different replications, the corresponding target MTP was cultivated and measured in the BioLector. The time to reach a scattered light intensity of 10000 arbitrary units (a.u.) was determined for each well and the time difference between the fastest and the slowest growing culture of each plate was determined. These time differences were used to compare the reproducibility of the transferred volume of the different replication tools. Most experiments were conducted once, whereas the experiment with the steel replicator with spring-loaded pins was done twice with time differences of 1.8 h and 2.1 h, indicating a sufficient reproducibility.

An *E. coli *pRHotHi-2-EcFbFP cryoculture was used to establish different initial biomass concentrations for an induction experiment. Therefore, the cryoculture was thawed, centrifuged and resuspended in MDG medium. Different amounts of this suspension were then used to establish different biomass concentrations (OD_600_: 0.0125, 0.025, 0.05, 0.1, 0.2) in a MTP. The cultures were grown and monitored in the BioLector. To induce the cultures, the cultivation was shortly interrupted after 4.9 h of cultivation, the sealing foil was removed and IPTG stock solution was added, resulting in a final IPTG concentration of 0.1 mM. Subsequently, the MTP was sealed with a new sealing foil and the cultivation was resumed. In previous studies this concentration was shown to be optimal for this strain in this specific medium [29].

### Simulation of a typical high-throughput cultivation process

**Figure 1:**
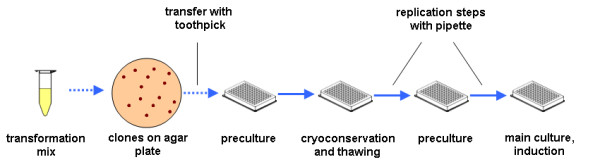
**Scheme of a typical high-throughput cultivation process**. After transformation, the clones are plated and grown on agar. Colonies are transferred with sterile toothpicks to a preculture-MTP and cultivated. Glycerol is added and the MTP is frozen (cryoculture). After thawing and mixing, liquid is transferred from the cryoculture-MTP to a new preculture-MTP. This MTP is incubated and subsequently a new main culture-MTP is inoculated with liquid from the preculture-MTP. At a defined point of time, inducer is added to the main culture to induce protein expression.

A typical high-throughput cultivation process is depicted in Figure 1. To simulate such a process, a MTP filled with 200 μL modified Eikmanns mineral medium per well was inoculated with 62 genetically identical *C. glutamicum *colonies from an agar plate with sterile toothpicks.

Then, the MTP was cultivated over night (14-16 h) at 37°C, a shaking frequency of 995 rpm and a shaking diameter of 3 mm. Subsequently, 20 μL glycerol stock solution (500 g/L) was added to each well of the MTP as a cryoprotectant, resulting in a final glycerol concentration of 45 g/L. The MTP was then frozen at -20°C. For the experiment, the cryoculture-MTP was thawed and mixed before 10 μL of each well were transferred to 190 μL modified Eikmanns mineral medium in a preculture-MTP with an 8-channel pipette. Subsequently, a precultivation was conducted over night (14-16 h). 10 μL of each well of this preculture-MTP were then transferred to 190 μL modified Eikmanns mineral medium in a main culture-MTP with an 8-channel pipette. After this, the corresponding MTP was cultivated and measured in the BioLector. The induction was conducted after 5.25 h of cultivation in the same way as described in the section Replication tools and experiments but with a final IPTG concentration of 0.5 mM.

## Results and Discussion

**Figure 2:**
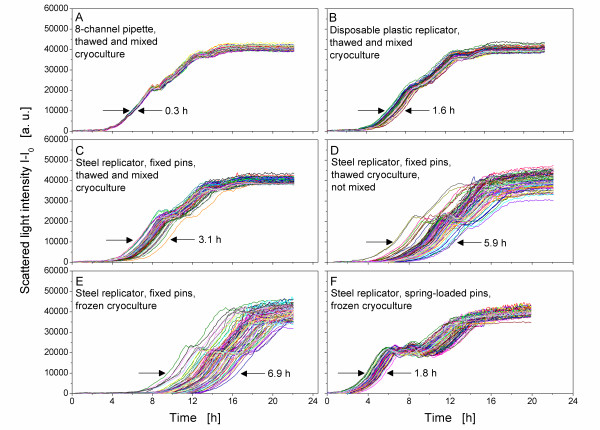
**Influence of different replication tools on growth of 96 cultures of *E. coli *PR02**. Replication tools: A) 8-channel pipette, thawed and mixed cryoculture. B) Disposable plastic replicator, thawed and mixed cryoculture. C) Steel replicator, fixed pins, thawed and mixed cryoculture. D) Steel replicator, fixed pins, thawed cryoculture, not mixed. E) Steel replicator, fixed pins, frozen cryoculture. F) Steel replicator, spring-loaded pins, frozen cryoculture. Experimental conditions: TB medium with 5 g/L glycerol, filling volume per well 200 μL, shaking frequency 950 rpm, shaking diameter 3 mm, temperature 37°C, measurement with BioLector.

A set of experiments was conducted to evaluate the influence of the replication step on growth kinetics of a single *E. coli*-strain (PR02). Cultures were transferred from a source cryoculture-MTP with 96 homogeneous cultures with identical biomass concentrations to a target MTP with different replication tools and replication methods. Figure 2A-F show the growth curves of these cultures in TB medium. A diauxic growth can be observed. The first growth phase on the carbon source glycerol ends with its depletion at a scattered light intensity of about 20000 a.u A second growth phase on complex compounds of the medium follows, resulting in a stationary phase at about 40000 a.u This growth pattern is consistent with the growth curves of *E. coli *BL21 in TB medium, as described by Kensy et al. [23]. The time difference between the fastest and the slowest culture for the particular replicator system was determined at 10000 a.u. as an indicator for the accuracy of the applied replication tools. While the curve progression of all six experiments (Figure 2A-F) is similar, their corresponding time difference of the fastest and slowest culture varies dramatically. Figure 2A-C depicts the time difference of *E. coli *PR02 cultures that were inoculated with different replication tools. All cryocultures were thawed and mixed before replication. The application of an 8-channel pipette leads to the lowest time difference (0.3 h). For the disposable replicator and the steel replicator with fixed pins, it is 1.6 h and 3.1 h, respectively (Figure 2B and 2C).

According to the manufacturer, the precision of the 8-channel pipette for a transfer volume of 5 μL is high with a coefficient of variance of only ≤ 2%. Higher transfer volumes increase the precision. In contrast to the 8-channel pipette, the transfer volume of the applied replicators is below 1 μL. In this low range, the volume is strongly affected by the manufacturing accuracy of the pins, leading to a lower precision of the replicator tools. This results in variances in transferred biomass and thus variable growth kinetics.

As depicted in Figure 2C-E, the time difference also varies strongly depending on the way the cryoculture-MTP is treated. Whereas thawing and mixing of the cryoculture-MTP results in a time difference of 3.1 h with the steel replicator (Figure 2C), merely thawing without mixing leads to a nearly doubled time difference of 5.9 h (Figure 2D). This might be explained by concentration gradients in the wells, caused by cell sedimentation during freezing of the cryoculture. When such a cryoculture is not mixed after thawing, the transferred amount of inoculum is obviously highly variable, leading to different growth kinetics. Besides concentration gradients also temperature gradients during freezing may influence the variability of cultures in MTPs, although the investigation of these aspects was not the focus of this study. To avoid the possible negative effects of temperature gradients, all cryoculture MTPs for the experiments of Figure 2 were frozen with the same procedure. Figure 2A demonstrates that a homogeneous growth pattern can be achieved indicating that the way to freeze and thaw the cryoculture-MTP is suitable to avoid variability.

The cultures that were inoculated without thawing the cryocultures have the longest time difference of 6.9 h (Figure 2E). As the pins of the applied steel replicator are fixed, their contact to the frozen liquid in the wells of the cryoculture-MTP varies, caused by slight differences of the height of the frozen liquid in each well. These differences can occur because of variable freezing patterns in the wells [28], resulting in varying filling heights. Thus, different amounts of liquid are thawed by the heat introduced by the pins and transferred to the target MTP, resulting in differing initial biomass concentrations. In contrast to these fixed pins, the pins of the other steel replicator are spring-loaded to equalize even small differences in the filling height of the wells. This grants a homogeneous contact of the pins to the surface of the frozen liquid in the wells of the cryoculture-MTP [28]. Thus, a reduced variance in transferred biomass in comparison to replicators with fixed pins was observed. This led to a time difference of only 1.8 h (Figure 2F) in comparison to 6.9 h for the steel replicator with fixed pins (Figure 2E). In summary, the variance in transfer volume is affected not only by the replication tool itself, but also by the replication method (e. g. mixing of thawed cryocultures). Furthermore, it is influenced by several other factors. According to a manufacturer of replicator tools, the transfer volume of pin replicators is, for example, affected by the speed of withdrawal of the replicator from the liquid as well as the source plate volume [30].

**Figure 3:**
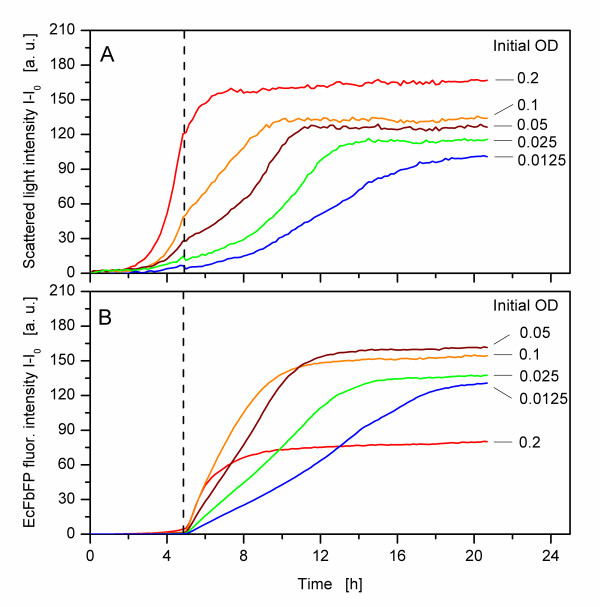
**Influence of different initial biomass concentrations on growth and product formation of *E. coli *BL21(DE3) pRhotHi-2-EcFbFP**. A) Scattered light intensity (mean of eight parallel wells per condition). B) EcFbFP fluorescence intensity (mean of eight parallel wells per condition). Vertical dashed line: time of induction with 0.1 mM IPTG after 4.9 h. Experimental conditions: MDG medium, filling volume per well 200 μL, shaking frequency 950 rpm, shaking diameter 3 mm, temperature 37°C, measurement with BioLector.

The effects of the different growth kinetics caused by the applied replication tools on product formation were studied with the strain *E. coli *BL21(DE3) pRhotHi-2-EcFbFP. This strain produces a model fluorescent protein upon induction with IPTG and thus allows the monitoring of the product formation with the BioLector. Figure 3 shows the growth and protein expression of *E. coli *BL21(DE3) pRhotHi-2-EcFbFP cultures with five different initial biomass concentrations, simulating the possible effects from replication steps. Because of the varying initial biomass concentration, different growth kinetics can be observed up to 4.5 h (Figure 3A). At that time, all cultures of the MTP are induced simultaneously with IPTG, which means that the cultures are induced at different physiological conditions. The cultures with an initial OD_600 _of 0.2, for example, are in the late exponential growth phase at the time of induction, whereas the cultures with an initial OD_600 _of 0.0125 are in the early exponential growth phase. With a final EcFbFP fluorescence intensity of about 80 a.u., the cultures with an initial OD_600 _of 0.2 have the lowest amount of product, whereas the highest amount of about 160 a.u. is reached by the cultures with an initial OD_600 _of 0.05 (Figure 3B). Apart from the different amount of synthesized product, the rate of product formation also varies. The cultures with an initial OD_600 _of 0.1 and 0.2 have the fastest rate of product formation, those with an initial OD_600 _of 0.0125 the slowest.

The reason for the different growth after induction is the metabolic burden the cultures are exposed to [31,32]. When the cells are induced, cellular resources are redirected to product formation, thus reducing the growth of the cultures. According to Donovan et al. [33], this burden influences the growth rate, cell yield and product expression of the culture. Furthermore, the amount of produced protein is dependent on the growth phase in which the cultures are induced. Therefore, if all cultures of a MTP are induced at the same time, which is a common procedure in high-throughput cultivation processes, differences in the initial biomass concentration lead to variances in product formation. Taking these variances into account, it seems impractical to screen and compare different clones regarding their product formation.

Differences in initial biomass concentration can be equalized by a robotic liquid handling system in combination with the BioLector (Robo-Lector) [29]. The Robo-Lector permanently monitors the preculture-MTP and calculates the amount of liquid that has to be transferred from each well of the preculture-MTP to the target MTP to reach homogeneous initial biomass concentrations ('biomass-specific replication'). A further possibility to equalize different biomass concentrations is to utilize silicone elastomer depots at the bottom of a MTP that release glucose during cultivation to establish fed-batch conditions [34]. In contrast to the aforementioned method that acts at the replication step from preculture to main culture, this fed-batch technique for MTPs can be applied at the precultivation step. Due to the fed-batch cultivation, all wells of a MTP are in the same growth phase and have equal biomass concentrations after a certain time of cultivation. Transferring an inoculum from such an equalized preculture to the main culture minimizes the differences in initial biomass concentrations.

Another way to deal with different initial biomass concentrations is not to equalize, but to compensate variable growth kinetics during the main culture. The Robo-Lector allows to induce single wells automatically when a defined biomass concentration is reached ('biomass-specific induction'). Thus, all screened cultures of a MTP are in the same growth phase and metabolic state when induced, resulting in an improved comparability between them [29]. Nevertheless, such a system can only be applied for medium-throughput cultivation processes, as it can currently monitor only one MTP.

**Figure 4:**
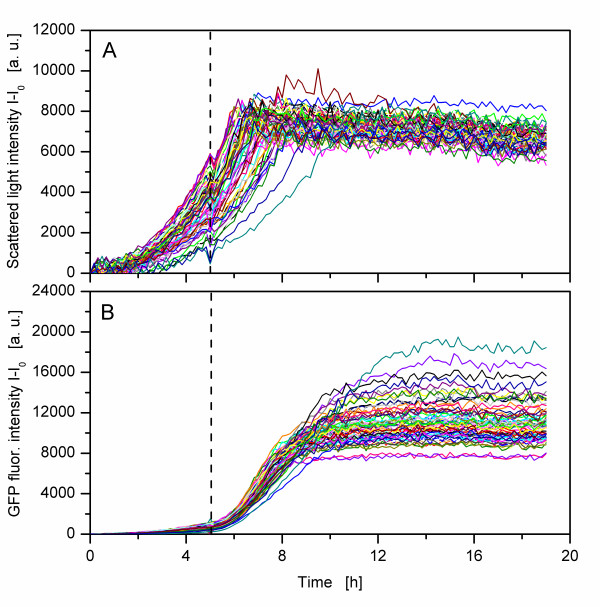
**Simulating a typical high-throughput cultivation process: effect on growth and product formation of *Corynebacterium glutamicum *pEKEx2-phoD-GFP**. Transformed genetically identical *C. glutamicum *clones were grown on agar-plate. Sixty-two colonies from this plate were transferred to a MTP with toothpicks and cultivated over night. Glycerine was added to the MTP which was then frozen at -20°C. The cryoculture-MTP was thawed and mixed. Subsequently, 10 μL of each well were transferred to 190 μL medium in a preculture MTP. After cultivation over night 10 μL of each well were transferred to 190 μL medium in a main culture MTP. The following cultivation with induction was monitored with the BioLector and is shown above. A) Scattered light intensity. B) GFP fluorescence intensity. Vertical dashed line: time of induction with 0.5 mM IPTG after 5.25 h. Experimental conditions: modified Eikmanns mineral medium, filling volume per well 200 μL, shaking frequency 995 rpm, shaking diameter 3 mm, temperature 37°C, measurement with BioLector.

Although such new techniques are emerging, the typical high-throughput cultivation process (as shown in Figure 1) with its multiple steps is a common procedure. In order to simulate such a high-throughput cultivation process, typical steps (clone picking, cryoculture, preculture, main culture and induction at a defined point of time) were conducted with *C. glutamicum*, another frequently applied and industrially important microorganism. Clones of *C. glutamicum *were transferred with sterile toothpicks from an agar plate to a preculture-MTP. After cultivation over night glycerol was added and the cultures were frozen. After thawing and mixing the cryoculture, another MTP was inoculated and a second preculture was conducted over night. Subsequently, a main culture-MTP was inoculated which was induced after 5.25 h for protein production. This last and most important step - the protein expression step - was monitored with the BioLector. For the replication step, an 8-channel pipette was applied which caused the lowest time difference in the first experiment (Figure 2A). Figure 4A shows the growth curves of 62 *C. glutamicum *pEKEx2-phoD-GFP cultures in a MTP. A strong variance can be seen between the different cultures. Whereas the fastest culture has a scattered light intensity of about 5500 a.u. after 5 h, the slowest culture reaches only about 1000 a.u When all cultures of the MTP are induced at a defined point of time (after 5.25 h in this study) variable product formation kinetics become visible (Figure 4B). The GFP fluorescence deviates by a factor of more than two, with a minimal GFP fluorescence signal of 8000 a.u. and a maximal GFP fluorescence signal of about 18000 a.u. at the end of the cultivation. The varying growth curves are presumably caused by varying initial biomass concentrations that lead, as a consequence, to strongly varying product formation upon induction.

As the replications were conducted with the highly accurate 8-channel pipette, the occurring strong variances can not be caused by the replication tool. It should be noted that cells were manually transferred with sterile toothpicks from an agar-plate to the first preculture MTP. The amount of inoculum transferred presumably varied strongly on each toothpick, leading to varying initial biomass concentrations. In addition, because of the manual picking, the inoculum was extracted from different regions of the single colonies (cell cluster) on the agar plate. Cells inside the cell cluster were probably in a different metabolic state, as the oxygen supply can be limited [35] and key nutritients can locally be depleted [36]. Hence, the transferred cells might not only have varied in their amount, but also in their metabolic state. Instead of manual tooth picking, more sophisticated techniques such as automated clone picking systems may be used [37]. Although these picking systems reduce time and workload for colony picking, the problem of varying cell concentrations and metabolic states is an inherent one. Cultures with a higher initial biomass concentration reach their stationary phase earlier and are, thus, longer in this phase. As the entrance into the stationary phase means morphological and physiological changes [38,39] this different length of the stationary phase might result in different physiological states of the cultures. Whenever such precultures are applied to inoculate another culture, this problem of different physiological states can occur. These differences can be carried over from the first preculture over the cryoculture and second preculture to the main culture. The initial biomass concentration in some wells might even have been too low to reach the stationary phase during precultivation. In that case, the cultures that were applied for inoculation might not only have differed in their physiological state, but also in their biomass concentration. Thus, although two precultures were conducted (one before and one after the cryoconservation), the variances in biomass concentrations caused by tooth picking were probably carried over to the main culture. Further studies should, therefore, monitor the whole process, beginning with the first preculture to investigate the influencing factors for such high-throughput cultivation processes.

## Conclusions

This study showed that the conventional method of using 96-pin replicators to transfer *E. coli *PR02-cells from one MTP to another MTP can result in highly variable growth kinetics of the applied cultures. To our knowledge, this problem could be demonstrated and quantified for the first time (with the help of the BioLector). The degree of variance of the growth kinetics depends on the applied replicator and replication method. It is caused by variances of the transferred liquid volume, resulting in varying initial biomass concentrations. In conclusion, replication tools can lead to unpredictable and irreproducible high-throughput cultivation processes. In contrast to the pin replicators, the 8-channel pipette showed nearly identical growth kinetics. Since there is a need for reliable and reproducible methods for the production of proteins in MTP-based high-throughput processes [40], applying liquid handling systems is strongly advised to reduce variances in transferred liquid volume.

Furthermore, growth and product formation of a simulated high-throughput process with *C. glutamicum *was monitored on-line with the BioLector technique. It showed that the typical steps of a high-throughput production process (colony picking, preculture, main culture and induction) can lead to strongly varying growth kinetics even if a defined single strain is investigated. These growth differences resulted in a strongly varying product formation upon induction of the main culture at a defined time. Additionally, this study showed that the initial biomass concentration strongly affects the product formation if all cultures of *E. coli *BL21(DE3) pRhotHi-2-EcFbFP in a single MTP are induced simultaneously. To sum up: high-throughput cultivation processes with multiple steps are prone to irreproducible protein expression results.

Different methods addressing the above problems have recently been developed. The combination of on-line monitoring (BioLector) with an automated liquid handling system can equalize varying growth kinetics by establishing homogeneous initial biomass concentrations in MTPs. This combination can also compensate varying growth kinetics and, hence, product formation, because it can automatically induce different cultures at a defined biomass concentration, in contrast to the manual induction at a defined time. Such compensation can also be achieved by the utilization of autoinduction medium or fed-batch precultures in MTPs. The presented methods and the awareness that varying initial biomass concentrations can occur during replication may minimize this problem and, therefore, might help to increase the robustness and reproducibility of high-throughput cultivation processes.

## Competing interests

The authors declare that they have no competing interests.

## Authors' contributions

RH designed the experimental setup and prepared the manuscript. TGP performed cultivation experiments and prepared the manuscript. NR, AKH, KL performed cultivation experiments. FK provided data for the *Corynebacterium *experiment. JB initiated the project, assisted with data analysis and manuscript preparation. All authors read and approved the final manuscript.
